# Superinfection exclusion studies using West Nile virus and Culex flavivirus strains from Argentina

**DOI:** 10.1590/0074-02760200012

**Published:** 2020-06-03

**Authors:** Silvina Goenaga, Julieta Goenaga, Estefanía Raquel Boaglio, Delia Alcira Enria, Silvana del Carmen Levis

**Affiliations:** 1 Instituto Nacional de Enfermedades Virales Humanas Dr Julio I Maiztegui, Pergamino, Buenos Aires, Argentina; 2 Universidad Nacional del Noroeste de la Provincia de Buenos Aires, Buenos Aires, Argentina; 3 Århus University, Århus Institute of Advanced Studies, Århus, Denmark

**Keywords:** superinfection exclusion, West Nile virus, Culex flavivirus, Argentina

## Abstract

In Argentina, many *Flavivirus* were recognised including West Nile virus (WNV). During 2009 several strains of Culex Flavivirus (CxFV), an insect-specific flavivirus, were isolated in the same region where circulation of WNV was detected. Hence, the objective of this study was to analyse the effect of co-infection *in vitro* assays using CxFV and WNV Argentinean strains in order to evaluate if CxFV could affect WNV replication. Our results showed that WNV replication was suppressed when multiplicity of infection (MOI) for CxFV was 10 or 100 times higher than WNV. Nevertheless, *in vivo* assays are necessary in order to evaluate the superinfection exclusion potential.

The genus *Flavivirus* (family *Flaviviridae*) comprises over 70 viruses that include several human pathogens such as Yellow fever, Dengue virus (DENV1-4), Zika virus (ZIKV) and West Nile viruses (WNV). Members of this genus are enveloped, positive-sense single-stranded RNA viruses. Their genome is approximately 11 kb in length, which includes a short 5´non-coding region (5´NCR), a single long open-reading frame encoding the structural proteins capsid (C), premembrane (prM,) envelope (E), seven non-structural proteins (NS1, NS2a, NS2b-NS3-NS4a-NS4b-NS5) and a 3´non-coding region (3´NCR). Based on their host associations, flaviviruses have been grouped into insect-specific flaviviruses (ISFV) which cause a persistent infection in the insect, and vertebrate viruses with either no known vector.^1^ Moreover, in the last decade novel insect-specific viruses, which are host-restricted to replication in invertebrate cells, were discovered in mosquitoes. These viruses phylogenetically affiliate with mosquito-borne flaviviruses (MBFV) despite their apparent insect-restricted phenotype.^2^

Superinfection exclusion (SIE) is the process by which host cells infected with one virus do not support productive replication of the same or similar virus.^3^ SIE assays with MBFV and ISFV have been performed in order to establish a preventative intervention strategy for blocking the transmission of agents of human diseases and in order to gain a better understanding of any additional factor(s) that could alter vector competence of mosquitoes in both enzootic and epizootic transmission cycles. In this sense, different *in vivo* and *in vitro* studies have assessed the relative potential of different ISFV to interfere with replication of flaviviruses of human health importance.^4^ ^,^ ^5^ ^,^ ^6^ ^,^ ^7^ ^,^ ^8^

In Argentina, several medically important MBFV have been detected in recent years.^9^ ^,^ ^10^ ^,^ ^11^ ^,^ ^12^ Since 1997, Argentina has experienced the re-emergence of DENV1-4, which represented a growing public health problem. Outbreaks caused by other flaviviruses, including WNV and St Louis encephalitis (SLEV) have also been reported in the country.^10^ ^,^ ^13^ More recently in 2016, another arbovirus named ZIKV were detected in humans in Tucumán, Salta, Chaco and Córdoba provinces (https://www.argentina.gob.ar/salud/boletines-epidemiologicos/2019/ Boletín integrado de vigilancia N434 SE1). Concerning WNV circulation, this virus was isolated in 2006 from the brains of three dead horses with clinical manifestations of encephalitis in Argentina. This was the first isolation of WNV in South America.^10^ After that, public health surveillance has been detected sporadic human cases from 2006-2007 in five provinces of the northeast and centre of Argentina (Chaco, Entre Ríos, Formosa, Santa Fé, and Córdoba provinces). However, the impact on animal and human public health was considerably lower than in the Northern hemisphere.^14^ Until now, there have been no reports of acute illness and most of human cases present mild symptoms.^14^ As Artsob et al.^14^ describes, this differential impact for WNV in both hemisphere may be attributable in part to higher species diversity with bird communities in the tropics, that includes many species that are poor amplifying hosts of WNV. On the other hand, there are numerous endemic and/or enzootic flaviviruses circulating in Argentina and South America, and this could provide cross-protective immunity in human and bird population.

The transmission cycle of WNV commonly involves birds and *Culex* mosquitoes, but it is not well known in Argentina. Interestingly, serological evidence of infection of WNV has been detected in monkeys in the same region where several strains of CxFV were isolated,^15^ suggesting that both flaviviruses are sympatric. This fact raised our interest to evaluate if CxFV may suppress replication of WNV, since as it was mentioned before, the impact of WNV on animal and human public health in Argentina is considerably lower than in the USA.

For that purpose, we performed co-infection *in vitro* assays, in order to evaluate the impact on WNV replication in co-infected cultures cells with different multiplicity of infection (MOI) for CxFV.

Co-infection studies were performed using WN-Eq001 and CxFV Otero2009 strains. WN-Eq001 strain (GQ379160) was isolated from the brain tissue of a horse with neurologic symptoms as described by Morales et al.^10^ The titer of WNV strain used was 7 log_10_ pfu/mL after three passages in Vero C76 cells. CxFV Otero2009 strain (KC700045) was isolated from *Cx. maxi* mosquito pool as described by Goenaga et al.^15^ The titer of CxFV Otero2009 strain used was 7.1 log_10_ pfu/mL after four passages in C6/36 cells.

In order to assess the *in vitro* potential of CxFV Otero2009 infection on WNV-Eq001 replication in the mosquito cell line, triplicate cultures of *Aedes albopictus* C6/36 cells in T_25_-cm^2^ flasks were inoculated. Three different tests were performed in order to evaluate the effect of CxFV Otero2009 infection on WN-Eq001 replication at different MOI ratio (Table).

**TABLE:** Co-infection assays of Culex Flavivirus (CxFV) Otero 2009 and WN-Eq001 strains

Test	Strain multiplicity of infection (MOI)	MOI ratio CxFV:WNV
Nº 1	CxFV Otero2009 (0,09) + WN-Eq001(0,09)	1:1
	WN-Eq001 (0,09) (control)	
Nº 2	CxFV Otero2009 (0,9) + WN-Eq001 (0,09)	10:1
	WN-Eq001 (0,09) (control)	
Nº 3	CxFV Otero2009 (0,9) + WN-Eq001 (0,009)	100:1
	WN-Eq001 (0,009) (control)	

Scheme of the test performed to evaluate co-infection effect of CxFV Otero2009 on WN-Eq001 replication in C6/36 cell line, at different MOI ratio. The assays were carried out by triplicate. WNV: West Nile virus.

Cell cultures in Dulbecco’s modified eagle medium (DMEM) supplemented with 10% foetal bovine serum (FBS), 1% L-glutamine and 1% penicillin streptomycin (growing cell media), were infected with 2 mL of a solution with both viruses in a proportion of MOI previously defined (Table). After 1 hour incubation at 34ºC, inoculum was removed and cells were -washed twice with phosphate-buffered saline (PBS) pH 7,4. Then, 8 mL of growing cell media was added. Viral controls were performed by infecting C6/36 cells with WN-Eq001 alone.

Aliquots of 200 uL of the cell-culture supernatants were collected immediately after infection and daily during 7 days. WNV titers were estimated by plaque forming units (pfu) titration on Vero C76 cells under agarose and were expressed as log_10_ pfu/mL. Titration was performed as previously described Medina et al.^16^ Briefly, tenfold serial dilutions of each sample in minimum essential medium (MEM) supplemented with 2% FBS and antibiotics were added in a confluent Vero C76 monolayers attached to 12-well plates and incubated for 1 hour with periodic gentle rocking to facilitate virus adsorption at 37ºC. The volume of the inoculums was 100 uL in each well. Plaques were incubated undisturbed for 5 days at 37ºC. Vital dye neutral red was used at 2% for plaque visualisation.

The mean WNV titers in cell culture at each day post-infection (dpi) were compared between cell cultures co-infected with CxFV Otero2009 + WN-Eq001 and control cultures infected with WN-Eq001 alone by analysis of variance (ANOVA) multiple comparison.

WNV growth curves shows at Figure (A, B, C). The peak titer of WNV replication was approximately at 5 dpi in both, the co-infected cells and the control cultures infected with WNV alone. However, WNV titers were significantly lower in the co-infection assays when inoculated with CxFV at an MOI 10 to 100 times higher than that used for WNV.

When CxFV: WNV MOI ratio was 10:1 (Test Nº2), WNV titer was significantly lower than that of the WNV control at all dpi, except at 2 dpi (F_1, 4_ = 161,18; p = 0,001) [Figure (B)]. Similar result were obtained when CxFV: WNV MOI ratio was 100:1 (Test Nº3); at all dpi (F_1, 4_ = 420,45; p < 0,001) [Figure (C)].

**Figure:**
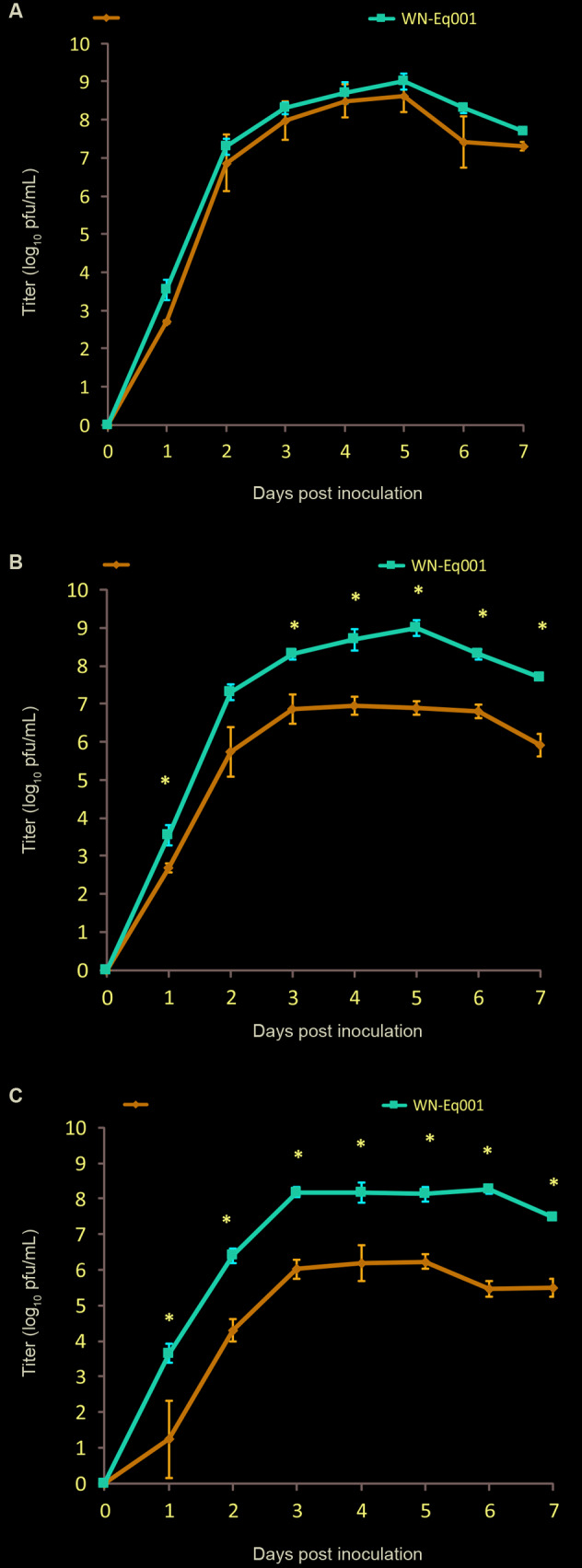
WN-Eq001 titers obtained from culture supernatants of C6/36 cells inoculated with Culex Flavivirus (CxFV) Otero 2009 and WN-Eq001 at different multiplicity of infection (MOI) ratio CxFV:West Nile virus (WNV) (panel A MOI ratio CxFV:WNV =1:1; panel B MOI ratio CxFV:WNV =10:1; panel C MOI ratio CxFV:WNV =100:1). Each data point represents the plaque titration mean of triplicate cultures. Bars represent standard deviations from the mean. *: significant difference. The limit of detection for WNV titers was 5 pfu/mL culture supernatant.

At Test Nº2, the peak WNV titers in co-infected cultures were 1,5 log_10_ pfu/mL or 2 log_10_ pfu/mL lower than those for cultures infected with WNV only at all dpi. Similar result are shown in Figure (C) (Test Nº3) where the peak WNV titers in co-infected cultures were ~2 log lower than those of cultures infected only with WNV at all dpi.

Finally, in C6/36 cells inoculated with CxFV and WNV at equal MOI (Test Nº1), no differences in WNV titers at all dpi between co-infected and solely-infected cultures were observed [Figure (A)].

In this study we demonstrated that co- infection of *A. albopictus* C6/36 cells with CxFV Otero2009 and WN-Eq001 interfere with WNV replication depending on the CxFV MOI used.

Our results showed a significant inhibition of WNV replication in presence of CxFV in earlier stages of infection, but only when MOI was 10 or 100 times higher for CxFV than for WNV. Bolling et al.^5^ evaluated sequential infections between CXFV and WNV in C6/36 cells. Infection with CxFV followed by WNV 48 h later, resulted in significantly reduced WNV titers in co-infected cells compared to controls, they also describe an inhibition for WNV replication in early stages of infection. A similar study performed by Kent et al.^4^ looking at WNV replication kinetics in C6/36 cells co-infected with CxFV demonstrated slightly reduced WNV titers, but these differences were not statistically significant. In contrast to what was observed in the present study with WNV, Kuwata et al.^8^ describe an increase of Japanese encephalitis virus (JEV) titer in presence of CxFV in dual infection assays with these viruses in a *Culex* cell line. However, it may be due to cellular lysis, given that JEV titer was correlated with a decrease of cell number in the cultures, suggesting that JEV titer increases because of cellular lysis. In our assays, we did not observe a correlation between cytopathic effects (CPE) and the MOI rate used. Moreover, at CxFV MOI of 10 and 100 [Figure (B, C, respectively)], the peak of WNV titer in co-infected cells was lower than observed at MOI rate 1:1 [Figure (A)], suggesting that CxFV MOI has an effect on WNV replication. Several authors evaluated the MOI effect using *in vitro* co-infection studies and different causes could explain this effect such as, an impediment for WNV virus to entry in the cell due to unequal competition by the membrane receptors that internalise the viruses, or by competition at the level of RNA synthesis.^17^ ^,^ ^18^ ^,^ ^19^

Pepin and Hanley,^20^ who evaluated the MOI effect using sylvatic and endemic DENV strains, concluded that there is a density-dependent effect that affects virus replication, possibly due to depletion of resources. In their experiment they demonstrated a suppression of replication when DENV MOI was five, probably because of competence of viral polymerase or other host factors, as suggested by Lohmann et al.^21^ Alternatively, viruses can compete for replication sites.^22^ This may be especially common in flaviviruses, which require binding to a specific membrane compartment.^23^ Probably, replication sites along the membrane could be saturated due to high levels of virus particles. Nevertheless, these results correspond to *in vitro* studies using different MOI, and this is not an explanation about what could happen in field mosquitoes. For that purpose, *in vivo* assays, using a colony of naturally infected mosquitoes, are necessary in order to evaluate the CXFV superinfection exclusion potential.

Only a few published studies have looked at the effects of insect-specific virus infection on vector competence for arboviruses using living mosquitoes.^4^ ^,^ ^5^ ^,^ ^7^ Regarding to CxFV, Kent et al.^4^ investigated vector competence of mosquitoes for WNV, comparing *Cx. quinquefasciatus* females experimentally infected with CxFV to uninfected mosquitoes. No significant effects on WNV replication were observed. Another study compared the vector competence for WNV using a *Cx. pipiens* colony naturally infected with CxFV and a colony of *Cx. pipiens* CxFV-free.^5^ The dually infected mosquitoes showed significantly reduced WNV dissemination rates at seven days post-infection.

On the other hand, a retrospective study on *Cx. pipiens* mosquitoes collected in Chicago, found that WNV-positive mosquito pools had a four-fold increased likelihood of also containing CxFV compared to WNV-negative pools.^24^ In contrast, Crockett and colleagues^25^ found no evidence to support an association between WNV and CxFV prevalence rates in *Cx. quinquefasciatus* populations in the southeastern United States.

The result of these studies suggests that experiments investigating interactions between insect-specific viruses and arboviruses may vary depending on: viral strains, mosquito species, geographic regions and their genetic variability, highlighting the need for additional studies to clarify these interactions. However, superinfection exclusion is just one mechanism by which a viral symbiont might alter the vector competence of a mosquito vector for an arboviral pathogen.^2^

The WNV replication inhibition observed by CxFV Otero2009 could explain the epidemiology of WNV in Argentina, given that WNV and CxFV were detected in sympatry in San Cayetano city (27º 34 ‘S, 58º 41’ W).^12^ ^,^ ^15^ It is likely that both flaviviruses are interacting in natural mosquito populations. However, since the results we present were carried out in *Ae. albopictus* cell lines, it will be interesting to carry out co-infection *in vivo* assays using *Culex* spp. mosquitoes, or *in vitro* assays using *Culex* cell lines. In Argentina, there are no reports of *in vivo* assays that evaluate the co-infection effect or SIE between CxFV and MBFV, and vector species of WNV remains unknown. Micieli et al.^26^ showed that argentinian *Culex* mosquitoes are moderately efficient vectors of WNV and less susceptible to infection than USA mosquito strains. However, it is worth mentioning that they tested WNV strains isolated in USA, and, as it was mentioned before, there are differences between the WNV infection epidemiology of the USA and Argentina. It will be interesting to perform vector competence and co-infection *in vivo* assay using both: argentinean WNV strain and *Culex* sp. mosquitoes. Many other factors could explain the epidemiology of WNV infection in Argentina such as lower virulence for South America WNV strains or genetic epidemiologic, and/or ecologic aspects.

Further studies are necessary to determine the role that CxFV play in nature and also to investigate interaction with other flaviviruses of medical importance.
